# Health Professions' Educators' Adaptation to Rapidly Changing Circumstances: The Ottawa 2020 Conference Experience

**DOI:** 10.15694/mep.2020.000047.1

**Published:** 2020-03-23

**Authors:** Judy McKimm, Trevor Gibbs, Jo Bishop, Paul Jones

**Affiliations:** 1 Swansea University; 2 AMEE; 3 Bond University

**Keywords:** Health professions educators, Digital learning, learning technologies, adaptation to crisis, conference organisation, change, COVID-19

## Abstract

This article was migrated. The article was marked as recommended.

Most health professions’ educators (HPEs) are used to responding to change, whether these are longer term organisational changes or short term crises, e.g. staff or student sickness or technical systems’ failures. Most of these changes, whilst they can be frustrating, typically have fairly straightforward and routine solutions. Other wider, environmental changes are also starting to affect educators, learners and the complex education and healthcare systems in which they operate, and these will have great impact in the relatively near future. However, it is the current crisis stemming from the global transmission of the coronavirus COVID-19 which has most recently impacted on HPE on a global scale.

Whilst many of us are very used to working virtually and using social media and other activities to work collaboratively, we still tend to rely on regular meetings with friends and colleagues (old and new) around the world at conferences and meetings. Similarly, most universities rely primarily on face to face teaching to provide their programmes, particularly in the early years. The COVID-19 pandemic has put all that into sharp relief, and many of us are having to make quick and sometimes reactive adaptations to our best-laid plans. In this article, we discuss some of our experiences from the recent Ottawa 2020 conference held in Kuala Lumpur from 1-5 March 2020, identifying some of the lessons learned that educators around the world will need to keep in mind as we move into what is currently unchartered territory. The learning lessons from our experience are that safety is paramount, communication and transparency is key; flexibility is needed from all stakeholders; technologies can help, but be realistic; acknowledge the need for psychological adaptation to change and crisis and tap into the wisdom and collegiality of the community. This paper specifically refers to Covid-19 but the learning lessons are applicable to other major challenges and the ideas described transferable to other situations.

## Introduction

Most health professions’ educators (HPEs) are used to responding to change, whether these are longer term organisational changes (e.g. changes in staffing, regulations or procedures) or short term crises, e.g. staff or student sickness or technical systems’ failures. Most of these changes, whilst they can be frustrating, typically have fairly straightforward and routine solutions. Other wider, environmental changes are also starting to affect educators, learners and the complex education and healthcare systems in which they operate, and these will have great impact in the relatively near future. However, it is the current crisis stemming from the global transmission of the coronavirus COVID-19 which has most recently impacted on HPE on a global scale.

Whilst many of us are very used to working virtually and using social media and other activities to work collaboratively, we still tend to rely on regular meetings with friends and colleagues (old and new) around the world at conferences and meetings. Similarly, most universities rely primarily on face to face teaching to provide their programmes, particularly in the early years. The COVID-19 pandemic has put all that into sharp relief, and many of us are having to make quick and sometimes reactive adaptations to our best-laid plans.

In this article, we discuss some of our experiences from the recent Ottawa 2020 conference held in Kuala Lumpur from 1-5 March 2020, identifying some of the lessons learned that educators around the world will need to keep in mind as we move into what is currently unchartered territory. This paper specifically refers to Covid-19 but the learning lessons are applicable to other major challenges and the ideas described transferable to other situations.

## Background and context

The authors are involved in various guises with AMEE (the Association for Medical Education in Europe) and in running the ESMELead course at international conferences and meetings. The course was due to run at the Ottawa 2020 conference, over four days prior to and throughout the conference, comprising a one-day pre-conference workshop, two in-conference lunchtime sessions, and a post-conference half-day. It typically recruits around thirty participants, all HPEs from many countries, including local attendees, all of whom have registered and paid for the course prior to the conference. The course has a mixture of leadership theory and group activities; it is very interactive and practical, involving three to four faculty from around the world, all of whom have experience and an academic background in leadership and management. All the course resources and materials are available on Moodle, which is the open source learning platform AMEE uses for its courses.

Planning for the course had been on-going for almost two years, and four faculty were going to be involved. However, 8 days before the conference was due to start, one of the faculty members from Canada stepped out as he was recovering from influenza and was concerned about travelling with coronavirus-type symptoms. Two days before their due travel date, the two key course developers and deliverers based in the UK were advised not to travel by their medical school. This led to a very swift exchange of messages between them, the one remaining local faculty member and the AMEE organisers in order to decide what to do. Given that the technology was available to run the course (or parts of it) remotely, we agreed that the two faculty in Wales would lead from there using Zoom ^TM^ and that two others (one stepping in at very short notice) would facilitate on the ground. We knew the time difference would require the UK team to work through the night, and we reorganised and restructured the course, so that the work was allocated according to participants’ needs and experience. This required moving some sessions around and not delivering others as they didn’t lend themselves to this blended approach.

This article is not a comprehensive ‘how to’ meet all challenges that educators might encounter, but aims to provide some ideas and suggestions gained through our experiences of having to respond to rapidly changing circumstances and stimulate further conversation.

### Learning lessons

• Safety is paramount, communication and transparency are key
• Flexibility is needed from all stakeholders
• Technologies can help, but be realistic
• Acknowledge the need for psychological adaptation to change and crisis
• Tap into the wisdom and collegiality of the community

## Safety is paramount, communication and transparency are key

One of the key responsibilities for educators who are planning and organising educational events, be these international conferences or local lectures, is the need to maintain safety during times of crisis or uncertainty. One mantra is: *hope for the best, prepare for the worst*, but we need to use some tools to do this. A risk analysis (e.g. Shuttleworth, 2017; MindTools, 2020) can be useful to give some indications as to the likelihood of certain things occurring and the possible impact if they do. This should be updated whenever the situation changes. On-going option appraisals (e.g. UK Government, 2017; UK Local Government, 2012) need to be undertaken as information flows in from multiple sources as to whether to cancel or postpone the event or provide the conference or learning in another form. Many HPEs also work clinically with vulnerable patients, therefore infection and transmission risks for delegates need to be considered, and all public health interventions possible need to be put in place to help protect them. Educators need to be able to utilise and draw on a range of appropriate decision-making tools and to understand how to appraise risks and options. One useful way of thinking about a complex situation where there is a lot of uncertainty is from a ‘whole brain’ perspective ( Herrmann, Herrmann-Nehdi, 2015) where we purposefully use all four quadrants of our brain, considering (in this order):

• Analytical - this is an analytical, logical, fact-based, quantitative approach - what do we **know**? what do we need to **know**?
• Practical - this is all about detailed organising, planning, scheduling, resourcing aspects - what might we need to **do?**
• Relational - this quadrant is about feelings, emotions, interpersonal relationships - how might people **feel**?
• Experimental - this holistic, big picture approach involves synthesising, integrating and using intuition - bringing all we have discussed together, what **should/could** we do?

In some cases, decisions will be taken out of our hands, for example if public health advice is to stop all gatherings, cancel flights from certain countries or close universities. In such cases, we need to mitigate the impact on learners and faculty alike. The timing of the Ottawa 2020 conference was difficult for the organisers because such decisions had not then been made, or made earlier, neither in the host country, nor in many of the countries from which educators would be travelling. Regular communications and transparency around available evidence providing answers to FAQs (frequently asked questions) to stakeholders are therefore vital to enable everyone to make decisions, which are as informed as possible. Having one central point of contact and for outgoing communications using different media is essential so that messages are up-to-date and consistent. Using social media (such as Facebook, Twitter, etc.) for rapid communication prior to the conference and throughout its duration, helps ensure messages are received by a wide range of people. Once conference delegates have registered for a conference, their future contact through conference websites is usually infrequent.

## Flexibility is needed from all stakeholders

In some cases, advice from governments or specific universities to their faculty will be to not travel, and, whilst this gives clarity to attendees, it leads to more uncertainty for organisers as the numbers of attendees (both speakers and delegates) becomes unclear. For a large conference, the logistics are complicated anyway, despite adding in such uncertainty, and so flexibility is needed on all sides regarding the schedule, accommodation, catering and technical support. The organisers will have to very quickly adapt their programme if speakers back out or numbers are much lower than planned. Providers of catering and other conference support may also have to acknowledge that things may change at short notice. Event insurance is essential; making sure it covers such disruptions. And faculty and delegates may also need to be flexible, by engaging in the conference remotely, stepping in to chair sessions or other activities, or to accept that not all the sessions they planned on attending are available in the format originally promoted.

For university students and their faculty, other issues arise if campuses are required to close at short notice, such as how to provide learning opportunities, run examinations, provide clinical placements and ensure students living away from home (especially international students) have accommodation and other facilities i.e. the technology, that will support online learning. Health professions’ programmes often run outside the typical university academic year, with their students having to meet requirements from the regulators as well as the university’s own regulations and this puts added pressure on educators and administrators alike. When health services are themselves stretched, shortages of suitable examiners, clinical teachers and supervisors and placements will quickly emerge. This has the potential to have huge impact on programmes and faculty, but primarily on the students themselves. Universities and schools will need to very quickly review processes, requirements and regulations around attendance, assessment and progression, decide how students can meet requirements, what steps can be taken to ameliorate the impact and which, if any, student groups to prioritise (e.g. final years who will form part of the workforce the following year). Effective and on-going communication between students, including student leaders and faculty is paramount, with ease of access systems in place. Regulatory bodies, employers and postgraduate training programmes may also have to review their processes and workforce planning arrangements, particularly if students cannot graduate on time, or in worst case scenario, at all. Obviously financial and reputational aspects need to be considered alongside the welfare of all those involved and this means some hard decisions will need to be made.

## Technologies can help, but be realistic

Around the world, we have heard how it has been assumed that schools and universities can simply switch to ‘online learning’. As educators, we know it is certainly not so simple, particularly when the learning is usually face-to-face or in clinical settings, when faculty aren’t used to or not trained to provide online or blended learning, when the support for such technologies is limited, or where the infrastructure is poor. However technologies can be useful for many forms of communication, including conference presentations, video lectures, podcasts, narrated slide shows and teleconsultations. Universities have turned to their Offices of Learning and Teaching to provide additional training sessions and support. Doctors and other health professionals will need to become more adept in providing care using remote and mobile technologies e.g. telehealth, and one of the impacts of COVID-19 in reducing social contact is that this is starting to be much more common.

At the Ottawa 2020 conference, a number of plenary presentations, workshops, oral presentations and courses were provided using Zoom™ and other communication technologies. This required rapid adaptation by the presenters, faculty ‘on the ground’ and delegates. For presenters of short ‘lecture’ type presentations, this is fairly straightforward, requiring some familiarity with the system, a good internet connection and skilful management at the ‘receiving end’ in terms of timing, questions and discussion. Clearly, there may be some issues with sound or visual quality, but generally people are receptive to having someone ‘beamed in’ to them. Time differences can be overcome with goodwill, with some presenters providing voice over PowerPoints (VOPS) for sessions (during which the participants’ microphones need to be muted), with in-room moderators fielding questions and disseminating comments. If these sessions have any interactivity, then this needs to be carefully managed to ensure that the online attendees feel part of the session.

Workshops and courses which are longer in duration and interactive in nature, with a combination of short presentations and activities, are more problematic. If the key facilitators are facilitating virtually, they may need to redesign their courses and materials, depending on the expertise, experience and availability of who is managing the ‘room’. It is essential to have facilitators on the ground who can manage group activities, moderate questions and discussion, and help organise timings of breaks. Using a backchannel communication, such as WhatsApp, is useful to keep in touch with the facilitators whilst not disrupting the whole room, with delegates largely unaware that in-action problem solving was taking place. The main issue for both facilitators and delegates is being able to sustain attention and interactivity for what might be seven or eight hours. From the transmitting end, it can be quite difficult keeping your own energy up and feeling engaged, especially if you are facilitating through the night or early morning when your body is saying you should be asleep. Participants need to feel engaged with both the virtual and present facilitators, so a careful mix of presentations (theory), activities and discussions is required. The addition of the presenter image box rather than a voice alone was welcomed by all attendees and allowed ‘connection’. It can sometimes be hard to manage lively discussions virtually with a big group, because of sound quality, so setting some ground rules around how this will be managed and regularly checking in as to how it is working is helpful “back in the room”, “back to Wales”.

## Acknowledge the need for psychological adaptation to change and crisis

Any change (even a positive one) involves some sort of loss and uncertainty, and a crisis (’real’ or perceived) which can stimulate a host of emotions including worry, anger or fear. Educators working in times of crisis or rapid change need to remember that people (including themselves) need some time to psychologically adapt to change. However, people respond very differently to situations and so thinking about how people might respond to situations is important, making sure communications are timely and as clear as possible and that certain ‘trigger’ words such as ‘crisis’ are only used when absolutely necessary.

People who are unable (or unwilling) to attend events because of COVID-19 might feel a mixture of emotions; relief that they are not putting themselves or others at potential risk, guilt at letting people down, or disappointment at not being able to engage with the event and meet up with colleagues and friends. If organisers can provide various opportunities for engagement, this can help people feel more empowered and in control, mitigate some of these feelings and maintain the involvement of the community. It is also important to celebrate the positive aspects of such changes. Being able to network and quickly form and extend existing relationships is vital, both to help people feel connected to a group or community and to form teams who can provide a rapid response to changing events. At the Ottawa 2020 conference, because some people were unable to attend, opportunities emerged for people to step up and do new things (with support), people worked with different teams to deliver courses and sessions, and, as educators, we adapted to engaging in learning and communications in different ways. Of course, these blended experiences are not the same as everyone meeting and engaging face to face, but COVID-19 and the climate emergency will require us to work very differently and by sharing our learning from such adaptations, we will be better equipped to meet and plan for an uncertain future.

## Tap into the wisdom and collegiality of the community

Our final learning lesson is to proactively tap into the wisdom and collegiality of the community in which you practice or work. One of the key things to emerge from the Ottawa conference was the way people rose up to work together from wherever they were to solve problems and make sure everyone was safe, yet had a great conference experience. This requires strong leadership and a willingness to bring the community together to lead activities to achieve common goals. The collective has a huge energy, willingness and power, and the more power we share, the more we have to use. The HPE community is bound together collegially with its shared values centred around improving healthcare and education through our practices and activities, and we have a huge shared wisdom and expertise which can be brought to bear in difficult times. Just as the world is sharing information and experiences about COVID-19, so must our communities establish means through which we can share ideas and best practices on a global, interconnected scale. This is essential in rapidly changing, complex situations to provide support for our community members and avoid making mistakes and reinventing the wheel. The uncertainty leading up to and throughout the conference reinforced the collegiality of the HPE community to ensure a successful conference for the organisers, in-room delegates, course participants plus virtual attendees.

## Post-Conference observations

There are longstanding discussions around the inequality of in-person conferences, as some academics are unable to travel due to parental or caring responsibilities or lack of funding. However, this pandemic has thrust everyone into considering what the future of HPE, conferences and training might look like. Post conference review and discussion with HPEs has allowed substantial reflection which will be invaluable to future teams. Conference organisers worked with the venue and determined the limitations and ensured support from the local University IT department was available. Clear principles were set to ensure quality of experience e.g. workshops were only permitted to go ahead if the reviewed plan was suitably supported by technology, and a facilitator who was comfortable and confident in running it with off-site peers. The quality experience was ensured by incorporation of new symposia with on-site delegates working together.

General themes reiterated that presenters had to consider how synchronous, blended face to face with online participation is very different to fully online or face to face. It allowed a leap of faith, a change and encouragement into the world of blended learning. The current disruption has allowed HPEs to consider the benefits of face to face versus, distance and modified blended learning, for example establishing regional hubs connected virtually for future conferences. It must be remembered however, that whilst benefits exist, it is important to plan these events carefully, as it can be exhausting to teach or facilitate for long periods using remote technologies. It can also be more of a strain on learners, so revisiting the balance between presentations, activities and breaks is needed. Here it can be useful to take a leaf from online instruction and ‘chunk’ the learning sessions/topics into fairly short time slots.

## Conclusion

The recent disruption reinforces that clear and transparent communication is paramount, and that HPEs who work collaboratively can achieve almost anything. The ‘whole brain’ perspective analytical, practical, relational and experimental can be utilised as preparation for and consideration of issues during times of change. As we finish writing, universities have cancelled face-to-face teaching, home working is being mandated, borders have closed and a 14-day quarantine is in place for returning travellers in many countries. Everyone, faculty and learners alike, needs to adapt to this uncertainty. As a community we must be ready to adapt, be prepared to share experiences and learn from past experiences of our colleagues e.g. New Zealander colleagues following the Christchurch earthquake and Singaporean colleagues following SARS. We have also realised, with scheduling and promotion in recent conferences and literature that HPEs wellbeing needs to be considered along with students as a whole university or community approach. During these times of uncertainty, anxiety is heightened; we must consider our own wellbeing and rely on peer support.

## Take Home Messages

Health professions’ educators are required to be adaptive, however the recent pandemic is impacting on all our activities. The lessons we learned from adapting to change at the Ottawa 2020 conference are as follows:

• Safety is paramount, communication and transparency are key
• Flexibility is needed from all stakeholders
• Technologies can help, but be realistic
• Acknowledge the need for psychological adaptation to change and crisis
• Tap into the wisdom and collegiality of the community

## Notes On Contributors

Professor Trevor Gibbs is an Independent Consultant and Professor of Medical Education and Primary Care, and Professor of Medical Education, First Affiliated Hospital, Sun Yat-sen University, Guangzhou, China. He is President of the Association for Medical Education in Europe (AMEE). ORCiD: https://orcid.org/0000-0002-1776-6518

Professor Judy McKimm is Professor of Medical Education and Director of Stategic Educational Development, and Programme Director, MSc in Leadership for the Health Professions at Swansea University, Wales. She is an ESMELead course facilitator. ORCiD: https://orcid.org/0000-0002-8949-5067

Associate Professor Dr Jo Bishop is Curriculum Lead for the MD Program and Associate Dean, Student Affairs and Service Quality at Bond University, Australia. She is an ESMELead course facilitator. ORCiD: https://orcid.org/0000-0003-2433-6572

Honorary Associate Professor Mr Paul Jones is Programme Director of the Graduate Entry Medicine (GEM) Programme at Swansea University, Wales. He is an ESMELead course facilitator.

## Declarations

Trevor Gibbs is President of the Association for Medical Education in Europe (AMEE). Judy McKimm, Jo Bishop and Paul Jones are all ESMELead course facilitators.
