# Supplementary material for: Health Professions' Educators' Adaptation to Rapidly Changing Circumstances: The Ottawa 2020 Conference Experience
Source: MedEdPublish (2016). 2020 Mar 23;9:47. [Version 1] doi: 10.15694/mep.2020.000047.1 (PMC10697444; doi:10.15694/mep.2020.000047.1)
Supplement: Peer review history

# Reviewer report: Reviewer response for version 1

**DOI:** 10.21956/mep.20023.r30564

**Published:** 2020-03-28

**Authors:** Madalena Folque Patricio

**Affiliations:** 1 School of Medicine, University of Lisbon

This review has been migrated. The reviewer awarded 5 stars out of 5 Congratulations, as like previous reviewers, I enjoyed very much reading your article, which is indeed timely and very well written. Let me take this opportunity to also congratulate the Organisers of the Ottawa Conference for what they have achieved. I fully agree that more than ever, health professions’ educators need to be ‘adaptive’. Ronald Harden, who with his great vision has been advocating changes that later prove necessary, has long advocated the need to move from 'standard uniform practice to an adaptive curriculum’. His words now take on a new dimension!The Take Home Messages, namely the call for health professions’ education built on safety, flexibility, realism, psychological adaptation as well as being based on wisdom and collegiality of the community, are extremely important. I dare to add two other dimensions, namely to have health professions’ educators pursuing Exigency and Excellence. As the future is uncertain, we must do our best when trying to achieve our objectives. Pursuing 'Excellence' in Teaching, as proposed by the AMEE ASPIRE Initiative, should guide us in this time of crisis, because ‘being just good’ may be not enough to cope with the consequences created by COVID-19. Thanks again for this very good article, which make us think.

Reviewer Expertise:

NA

No decision status is available

# Reviewer report: Reviewer response for version 1

**DOI:** 10.21956/mep.20023.r30562

**Published:** 2020-03-27

**Authors:** P Ravi Shankar

**Affiliations:** 1 American International Medical University

This review has been migrated. The reviewer awarded 4 stars out of 5 I enjoyed reading this interesting article about the 2020 Ottawa Conference. With recent events online learning is becoming more common. The challenges of facilitating small groups online have been well described by the authors. The adaptability of the organizers, the faculty/facilitators and delegates were important to the success of the conference under rapidly changing circumstances. I would be interested in knowing greater details of how facilitators on the ground interacted with the remote faculty to ensure the smooth conduct of activity-based sessions. The authors have mentioned about the use of Whatsapp. A similar format is increasingly being considered globally. Sri Balaji Vidyapeeth in Puducherry, India is conducting educational and other sessions using a similar model of using local faculty or facilitators and remote experts leading the sessions online. This I believe is the first of a possible series of papers on the impact of the COVID-19 pandemic on teaching-learning activities and the response of health professions educators to this alarming crisis.

Reviewer Expertise:

NA

No decision status is available

# Reviewer report: Reviewer response for version 1

**DOI:** 10.21956/mep.20023.r30563

**Published:** 2020-03-27

**Authors:** Katharine Boursicot

**Affiliations:** 1 Duke-NUS Medical School

This review has been migrated. The reviewer awarded 5 stars out of 5 Excellent and timely paper in a time of a difficult global pandemic situation.The authors describe an innovative approach to delivering a course/workshop, leveraging modern technology. Additionally, they base the paper with sound theoretical underpinnings.A very helpful description of a novel approach to delivering teaching and learning under unusual circumstances.

Reviewer Expertise:

NA

No decision status is available

# Reviewer report: Reviewer response for version 1

**DOI:** 10.21956/mep.20023.r30558

**Published:** 2020-03-27

**Authors:** Puja Dulloo

**Affiliations:** 1 Pramukhswami Medical College

This review has been migrated. The reviewer awarded 5 stars out of 5 Reading this paper encouraged my thought, when such a big conference could be well organized during the lockdown state of Covid-19 pandemic, then I can take my online session for 150 students. Timely published article, to overcome the educational challenges during the hour of national lockdown with a decrease in personal socialization, and increase in technology based socialization. I enjoyed reading this article, what I take away from this article is that health professional educators have ability to adapt to change and are capable to overcome the challenge in empathetic and humanely manner, looking into the feelings of the other stakeholders. Moreover, allowed me to reflect, so as to improvise my teaching learning skills for better use of technology (outside the classroom setting) and have technology based networking and collaboration.

Reviewer Expertise:

NA

No decision status is available

# Reviewer report: Reviewer response for version 1

**DOI:** 10.21956/mep.20023.r30557

**Published:** 2020-03-26

**Authors:** Subha Ramani

**Affiliations:** 1 Harvard Medical School, Brigham and Women's Hospital

This review has been migrated. The reviewer awarded 5 stars out of 5 I read this paper with great interest as I attended the Ottawa conference with circumstances changing rapidly. I saw how effectively online presentations were organized- live and recorded- almost at the last minute. The leaders of the organization and presenters responded to the crisis as a community of educators and were flexible in adopting new formats to their teaching.This conference could be a great guide to a blended educational format for future conferences. The value of the in-person element can never be minimized, but certain content is ripe for online formats.Congratulations to the conference organizers for responding so well to a global threat and providing a sense of community.

Reviewer Expertise:

NA

No decision status is available

# Reviewer report: Reviewer response for version 1

**DOI:** 10.21956/mep.20023.r30560

**Published:** 2020-03-26

**Authors:** David Taylor

**Affiliations:** 1 Gulf Medical University, Ajman, UAE

This review has been migrated. The reviewer awarded 5 stars out of 5 This is an interesting and well written paper which shows us the direction that many international meetings are likely to be following. Like several other reviewers of this paper I was present at the Ottawa conference, and spoke with presenters and participants, who all attested to the success of the venture. Like many people, I think that face-to-face interactions are more fun, and probably more effective, but we are now in a world where travel and time away are likely to be restricted for the foreseeable future. The take home messages are going to be applicable to almost everything we do in the future.

Reviewer Expertise:

NA

No decision status is available

# Reviewer report: Reviewer response for version 1

**DOI:** 10.21956/mep.20023.r30565

**Published:** 2020-03-25

**Authors:** Pat Lilley

**Affiliations:** 1 AMEE

This review has been migrated. The reviewer awarded 5 stars out of 5 I enjoyed reading this paper, which is likely to be one of many that report educators’ experiences of, and responses to, the need to change teaching and learning practices as a result of the Covid-19 pandemic. Whilst this case study relates specifically to the ESMELead course delivered remotely by two of the authors based in the UK to face-to-face participants at the Ottawa 2020 Conference in Kuala Lumpur, the lessons learned from the course delivery are generalisable to teaching and learning more widely. What I take away from this paper is the overwhelming message that health professions educators are adaptable, up for a challenge, immensely professional and empathetic, and that much can be achieved through collaboration.

Reviewer Expertise:

NA

No decision status is available

# Reviewer report: Reviewer response for version 1

**DOI:** 10.21956/mep.20023.r30559

**Published:** 2020-03-24

**Authors:** Trudie Roberts

**Affiliations:** 1 Leeds Institute of Medical Education

This review has been migrated. The reviewer awarded 5 stars out of 5 A timely and excellent piece on meeting the educational challenges of our current global pandemic. Even after some levels of normality return this experience and advice will have relevance as the authors point out.I was at the Ottawa conference where this course was run and I know from speaking to several of the participant how much they enjoyed the course and appreciated the effort the presenters went to to make it a success. I recommend this as important reading for education in the current situation

Reviewer Expertise:

NA

No decision status is available

# Reviewer report: Reviewer response for version 1

**DOI:** 10.21956/mep.20023.r30561

**Published:** 2020-03-23

**Authors:** Felix Silwimba

**Affiliations:** 1 University of Lusaka

This review has been migrated. The reviewer awarded 5 stars out of 5 this article is well timed and has raised issues affecting many universities and the HPEs. recently my university closed down and drove us into panic state. it is a private university dependant on student user fees to run and pay salaries. so the uncertainity has just worsened. we do not know how long the shut down will last. therefore, the article is relevant to all regions of the world.

Reviewer Expertise:

NA

No decision status is available
